# Influenza Vaccination: A Cross-Sectional Survey of Knowledge, Attitude and Practices among the Lebanese Adult Population

**DOI:** 10.3390/ijerph121215000

**Published:** 2015-12-05

**Authors:** Ghada El Khoury, Pascale Salameh

**Affiliations:** Department of Pharmacy Practice, School of Pharmacy, Lebanese American University, PO Box: 36-Byblos, Lebanon; pascale.salameh@lau.edu.lb

**Keywords:** influenza, vaccination, immunization, Lebanon

## Abstract

*Background*: Influenza is a common preventable infectious disease associated with high mortality and morbidity. Vaccination is the most cost-effective measure to prevent influenza, yet the vaccine uptake is known to be low. No previous studies have assessed the rate of seasonal influenza vaccination use among the Lebanese population, nor examined the knowledge and attitudes towards the influenza vaccine. *Methods*: A cross-sectional survey was performed in 30 pharmacies randomly selected across Lebanon. A 19-item questionnaire was used to record influenza vaccination status, knowledge and attitudes towards the influenza vaccine among the Lebanese general population. *Results*: The survey response rate was 93%. Among the 640 study participants, the overall 2014-2015 seasonal influenza vaccination rate was 27.6%. The majority of participants (72.4%) reported irregular uptake of the vaccine. Results of the multivariate analysis revealed that elderly people (OR = 2.25, CI = 1.08–4.71), with higher education (OR = 1.42, CI = 1.09–1.84), higher physical activity (OR significantly higher than 1 for all categories), and chronic respiratory disease (OR = 3.24, CI = 1.58–6.62) were more regularly vaccinated, while those who visit the doctor “only when needed” (OR = 0.55, CI = 0.34–0.88) and those who consume more than seven drinks/week (OR = 0.24, CI = 0.09–0.65) were less regularly vaccinated. When introducing knowledge and attitude variables to the model, “thinking that the vaccine was not needed” was the only correlate that demonstrated a significant inverse association with regular influenza vaccination (OR = 0.15; *p* = 0.017). *Conclusions*: Suboptimal vaccination rates exist among the Lebanese ambulatory adult population. Clear misinformation on the importance of regular influenza immunization is also highlighted. This evidence underscores a compelling need to raise public awareness regarding the efficacy of the influenza vaccine.

## 1. Introduction

The influenza A and B viruses are human respiratory viruses that spread easily from person to person by droplets and aerosols [1]. Both viruses can affect any person in any age group and can cause seasonal influenza epidemics. Influenza A virus has the additional ability to generate worldwide pandemics. The Spanish flu (1918), Asian flu (1957), Hong Kong flu (1968) and the 2009 global outbreak of H1N1 that evolved into a seasonal pattern in 2010, were associated with millions of deaths worldwide [1]. Each year, epidemics of seasonal influenza also result in about 3–5 million cases of severe illness and 250,000–500,000 deaths worldwide [1]. Moreover, seasonal influenza triggers lost workforce productivity and strain health services and is therefore considered a serious public health problem. Influenza vaccination is the most effective way to prevent infection and severe outcomes like hospitalization, mortality and morbidity [1,2,3]. For instance, vaccination in the elderly was shown to decrease the risk of death from pneumococcal diseases and influenza-related complications by 50% and 80%, respectively [4].

The latest recommendations from the World Health Organization (WHO) and the U.S. Center for Disease Control and Prevention (CDC) state that individuals aged 6 months and older are encouraged to get vaccinated against influenza in attempt to expand protection to more people. It is particularly important for individuals in high-risk groups to receive the annual influenza vaccination to prevent the risk of serious complications [5,6]. High risk groups are defined as individuals highly exposed to the influenza virus and or highly prone to develop severe disease or death post-exposure [5]. Pregnant women, pediatric and elderly patients, as well as patients with chronic heart or lung diseases, metabolic diseases and immunosuppressed states are classified as high risk [5,6]. Despite clear evidence on the benefits of the vaccine, adherence to the issued recommendations is not always successful. The CDC estimates annual influenza vaccination coverage for the United States by utilizing data from several nationally representative surveys. For the 2014/2015 seasonal influenza, it was revealed, as of early November, that more than half of Americans had not yet received the recommended influenza vaccination [7]. The European CDC published a report in 2014 that included the annual surveys conducted by the Vaccine European New Integrated Collaboration Effort (VENICE) project. The report concluded that only a handful of European Member States were achieving the influenza vaccination specific targets as per the recommendations [8]. 

In Lebanon, influenza vaccine is paid for out-of-pocket since it is not part of the national vaccination program. Moreover, no national surveillance programs or awareness campaigns for influenza exist in the country. Data on the uptake of the influenza vaccine, knowledge and attitudes of the Lebanese population towards it, lack. A study by Romani *et al.* looked at the knowledge and beliefs of Lebanese family physicians regarding influenza vaccines and reported a clear deficiency in immunization awareness and practices [9]. In light of this, examining vaccination coverage rates as well as understanding the beliefs regarding influenza vaccine among the Lebanese community is an essential first step to postulating recommendations on vaccination practices. The objective of the proposed study is to evaluate the 2014–2015 seasonal influenza vaccination rate as well as assess the knowledge, attitudes and beliefs among a select Lebanese population towards the seasonal influenza vaccine. 

## 2. Methods

A quantitative cross-sectional research design was developed to study the influenza vaccination rate among the Lebanese general population as well as the attitudes, and knowledge towards the influenza vaccine. The study was approved by the Lebanese American University’s Institutional Review Board. The purpose of the study was elucidated and participant consent was obtained prior to administering the surveys. 

### 2.1. Sample

In Lebanon, pharmacies are considered easily accessible and constitute important frontline healthcare facilities for ambulatory patients, as the primary care services are relatively weak with only around 90 certified family physicians practicing in the country [9]. Therefore, the targeted population of ambulating Lebanese patients aged 18 years or above, was recruited from a random sample of community pharmacies across different regions of Lebanon from July to September 2015. To ensure a representative sample of adults, 30 pharmacies were randomly selected across the six Lebanese governorates: North of Lebanon, Mount Lebanon, Beirut, Beirut Suburb, South of Lebanon and Beqaa. In each pharmacy, around 20 subjects were interviewed face-to-face by pharmacy interns and were asked about their vaccination status. Since community pharmacies do not keep medical records, patients’ vaccination status, their knowledge and attitudes towards the influenza vaccine were self-reported through a questionnaire. 

### 2.2. Instrument

A 19-item questionnaire was developed based on available relevant literature. The instrument was used to record influenza vaccination status for 2014–2015 and assessed vaccination history. Yearly vaccine recipients were labeled as the “regular influenza vaccination” group and individuals who never or occasionally received the vaccine were the “irregular influenza vaccination” group. Knowledge and attitudes towards the vaccine among the Lebanese population were also collected.

The instrument included six parts: socio-demographics (gender, age, region, educational level, financial situation, health insurance, smoking, alcohol consumption, and physical activity), medical history (frequency of medical visits, medical conditions, medicinal drugs and indications), vaccination status (date of last vaccine received in general and influenza vaccine in particular), general knowledge of seasonal influenza. Interviewed individuals were asked if they were able to recognize the influenza symptoms, its complications, its high risk groups and its vaccine. Awareness variables were assessed as binary outcomes. Willingness to get vaccinated, the perceived barriers and vaccine information sources (physician, pharmacist, family and friends, religious groups, media, governmental agencies) were also self-reported. To evaluate the clarity of the survey queries, a pilot study was carried out on 20 volunteers. Accordingly, minor modifications were made to question wording and layout based on feedback from the respondents and interviewers.

### 2.3. Data Analysis

Data was entered and analyzed using SPSS software, version 22.0 (IBM Corp, Armonk, NY, USA). We used descriptive statistics to assess socio-demographic characteristics, basic knowledge, as well as perceptions especially with regard to safety and usage, and practices. Multivariable logistic regression was used to assess associations of regular vaccine use among the interviewees as a dependent variable with independent variables, using adjusted odds ratios (OR). Socio-demographic factors, current health status, and participants’ attitudes towards the vaccine were the independent correlates and potential confounders. *P* values < 0.05 were considered statistically significant.

## 3. Results

### 3.1. Sample Description

A total of 640 surveys were collected. The highest percentage (43%) of participants resided in Mount Lebanon (one of the major Lebanese governorates), 29.2% resided in Beirut and suburbs (the capital), while the rest resided in other Lebanese regions. Among the 640 study participants, 299 (46.7%) were males, participants age ranged between 18 and 88 years and the median age was 33 years. The sample distribution was judged to be representative of the Lebanese population in terms of age and gender, when compared to Lebanon official statistics (*p* > 0.05) [10]. Concerning the education level, 361 (56.4%) had a university degree, 181 (28.3%) reached high school, while 14.7% had low levels of education. The majority of participants (56.9%) were employed and 9.7% declared having no medical insurance. Our sample included patients belonging to high risk groups; 54 patients (8.4%) were aged 65 years or more, 75 (11.7%) had diabetes, 73 (11.4%) suffered from chronic heart disease, 40 (6.3%) had chronic respiratory disorder, 10 (1.6%) had cancer, 10 (1.6%) 9 (1.4%) chronic kidney disease and six (1%) were on chronic steroid therapy.

### 3.2. Rates of Vaccine Uptake

In this study, the overall 2014–2015 seasonal influenza vaccination rate was 27.6%. The majority of participants (72.4%) reported regular uptake of the vaccine. Among elderly patients aged more than 65, 35.2% received regularly the vaccine. Except for respiratory disease, the majority of patients with chronic diseases did not report receiving immunization against influenza. For instance, 79.5% of patients with heart disease, 81.3% with diabetes, 81.8% with cancer and 83.3% of patients on chronic steroid therapy did not receive seasonal influenza vaccination.

**Table 1:** Socio-demographic characteristics and regular vaccination.

Characteristic	Regular Influenza Virus Vaccination 176 (27.6%)	Irregular Influenza Virus Vaccination N = 461 (72.4%)	*p*-Value
Gender			
Male	75 (25.1%)	224 (74.9%)	0.177
Female	101 (29.9%)	237 (70.1%)	
Age quartiles			
<24 years	50 (30.7%)	113 (69.3%)	0.029
24–33 years	51 (32.3%)	107 (67.7%)	
34–51 years	40 (23.8%)	128 (76.2%)	
52–64 years	16 (16.8%)	79 (83.2%)	
65 and more	19 (35.2%)	35 (64.8%)	
Area of residency			
North Lebanon	20 (27.8%)	52 (72.2%)	0.366
Mount Lebanon	85 (30.9%)	190 (69.1%)	
Beirut	45 (24.1%)	142 (75.9%)	
Beirut suburbs	11 (18.6%)	48 (81.4%)	
South Lebanon	8 (32.0%)	17 (68.0%)	
Bekaa	2 (22.2%)	7 (77.8%)	
Education level			
No education	4 (11.1%)	32 (88.9%)	0.004
Below high school	17 (29.3%)	41 (70.7%)	
High school	38 (21.0%)	143 (79.0%)	
University degree	117 (32.4%)	244 (67.6%)	
Employment			
No	69 (25.4%)	203 (74.6%)	0.294
Yes	106 (29.1%)	258 (70.9%)	
Health Insurance			
None	12 (19.4%)	50 (80.6%)	0.008
Public insurance	55 (22.4%)	190 (77.6%)	
Private insurance	107 (32.7%)	220 (67.3%)	
Financial situation			
Comfortable	106 (32.7%)	218 (67.3%)	0.007
Manageable	60 (23.0%)	201 (77.0%)	
Difficult	8 (16.7%)	40 (83.3%)	

### 3.3. Association of Participants Socio-Demographic, Lifestyle Characteristics and Seasonal Influenza Vaccination

The correlations between socio-demographic and lifestyle characteristics and regular vaccination are summarized in Table 1 and Table 2, respectively. Education levels, employment status as well as perceived financial situation affected vaccination uptake. There was a clear and significantly low level of vaccination among those with a low level of education compared to more educated individuals (*p* = 0.004). Additionally, participants with no health insurance and with public insurance had a lower level of vaccination in comparison with private insurance (*p* = 0.008); moreover, people with perceived “comfortable” financial situation had higher level of vaccination (*p* = 0.007). Gender and area of residency did not affect vaccination rates, while age affected it. A lower rate of vaccination was observed with increased age, except for patients aged 65 and above who showed higher rates of regular vaccination *vs.* younger patients (*p* = 0.029). Regarding social history, the highest rates of influenza vaccines were found among non-smokers and heavy smokers (*p* = 0.03). Heavy alcohol consumers presented the lowest rate of vaccination (*p* = 0.013), as well as participants reporting to never perform any physical activity (*p* = 0.001). Moreover, subjects who reported medical visits “only when needed” as opposed to routine medical visits had the lowest rate of influenza vaccination (*p* = 0.001) 

**Table 2:** Lifestyle characteristics and influenza vaccination.

Characteristic	Regular Influenza Virus Vaccination 176 (27.6%)	Irregular Influenza Virus Vaccination N = 461 (72.4%)	*p*-value
Cigarette smoking			
Never	115 (31.5%)	250 (68.5%)	0.030
<15 cigarettes/day	40 (21.6%)	145 (78.4%)	
16–24 cigarettes/day	13 (19.7%)	53 (80.3%)	
25 cigarettes/day or more	7 (36.8%)	12 (63.2%)	
Alcohol drinking			
Never	86 (27.5%)	227 (72.5%)	0.013
1–7 drinks/week	85 (30.6%)	193 (69.4%)	
More than 7 drinks/week	5 (10.2%)	44 (89.9.2%)	
Exercise			
Never	50 (19.3%)	209 (80.7%)	0.001
Less than twice/week	53 (29.9%)	124 (70.1%)	
2–3 times/week	50 (35.7%)	90 (64.3%)	
More than 3 times/week	23 (37.7%)	38 (62.3%)	
Medical visit			
Routinely	46 (33.8%)	90 (66.2%)	0.001
Once/year	37 (38.5%)	59 (61.5%)	
When needed	91 (22.8%)	309 (77.3%)	

### 3.4. Association of Chronic Disease Conditions and Influenza Vaccination

When observing the vaccination rates among patients with specific comorbidities, there was no significant association established between chronic disease conditions and influenza vaccination, except for chronic respiratory diseases. Patients with respiratory diseases were found to have almost a double rate of seasonal influenza vaccination when compared to patients without this comorbidity (50% *vs.* 26.3%, *p* = 0.001) as shown in Table 3. 

**Table 3:** Chronic disease conditions and influenza vaccination.

Characteristic	Regular Influenza Virus Vaccination 176 (27.6%)	Irregular Influenza Virus Vaccination N = 461 (72.4%)	*p*-Value
Heart disease			0.144
Yes	15 (20.5%)	58 (79.5%)	
No	161 (28.7%)	400 (71.3%)	
Neurological disease			0.735 *
Yes	2 (20.0%)	8 (80.0%)	
No	173 (27.8%)	450 (72.2%)	
Diabetes mellitus			0.061
Yes	14 (18.7%)	61 (81.3%)	
No	162 (29.0%)	397 (71.0%)	
Liver disease			1.000 *
Yes	1 (33.3%)	2 (66.7%)	
No	175 (27.7%)	456 (72.3%)	
Kidney disease			0.069 *
Yes	0	9 (100.0%)	
No	176 (28.2%)	448 (71.8%)	
Cancer			0.736 *
Yes	2 (18.2%)	9 (81.8%)	
No	173 (27.9%)	447 (72.1%)	
Respiratory disease			0.001
Yes	20 (50.0%)	20 (50.0%)	
No	156 (26.3%)	438 (73.7%)	
Disease requiring steroids treatment			1.000 *
Yes	1 (16.7%)	5 (83.3%)	
No	174 (27.8%)	452 (72.2%)	

* Fisher exact test used.

### 3.5. Knowledge, Attitude and Practice of Influenza Vaccination

In Table 4, we present associations between knowledge, attitude and practice of influenza vaccination. A better knowledge about the influenza disease (symptoms, severity and risk) and about the vaccine (availability, efficacy and safety) is associated with higher regular vaccination rate (*p* < 0.001). When assessing the vaccine source of information, we found that the pharmacist (*p* = 0.001) and the physician (*p* = 0.024) followed by family members (*p* = 0.025) are associated with higher regular vaccination rates. The media (*p* = 0.158) and the government (*p* = 1) were not shown to have any role in disseminating information on the influenza vaccine.

**Table 4:** Knowledge, attitude and practice of influenza vaccination.

Characteristic	Regular Influenza Virus Vaccination 176 (27.6%)		*p*-Value
Aware of influenza symptoms			<0.001
Yes	168 (30.8%)		
No	6 (9.1%)		
Aware of influenza severity			<0.001
Yes	149 (34.3%)		
No	19 (12.8%)		
Aware of influenza risk			<0.001
Yes	144 (37.8%)	237 (62.2%)	
No	20 (10.8%)	166 (89.2%)	
Aware of vaccination needs/availability			0.001
Yes	170 (29.8%)	401 (70.2%)	
No	5 (11.9%)	37 (88.1%)	
Thinks that vaccination is effective			<0.001
Yes	145 (43.2%)	191 (56.8%)	
No	11 (10.3%)	96 (89.7%)	
Does not know	18 (9.5%)	171 (90.5%)	
The vaccine should be taken at a specific time			<0.001
Yes	146 (42.0%)	202 (58.0%)	
No	11 (13.6%)	70 (86.4%)	
Does not know	16 (7.8%)	188 (92.2%)	
Thinks the vaccine is safe			<0.001
Yes	155 (38.8%)	244 (61.2%)	
No	10 (16.9%)	49 (93.1%)	
Does not know	9 ( 5.1%)	166 (94.9%)	
Thinks the vaccine is for children only			<0.001
Yes	16 (23.9%)	51 (76.1%)	
No	144 (33.1%)	291 (66.9%)	
Does not know	15 (11.3%)	118 (88.7%)	
Has a fear of needles			0.720
Yes	3 (4.8%)	59 (95.2%)	
No	8 (3.9%)	198 (96.1%)	
Thinks the vaccine is expensive			0.371
Yes	0	32 (100%)	
No	11 (4.7%)	223 (95.3%)	
Thinks there is no need for the vaccine			0.127
Yes	4 (2.7%)	145 (97.3%)	
No	8 (6.5%)	115 (93.5%)	
Source of information is the physician	117 (31.0%)	260 (69.0%)	0.024
Source of information is the pharmacist	109 (33.0%)	221 (67.0%)	0.001
Source of information is the parents/family	40 (21.5%)	146 (78.5%)	0.025
Source of information is the media	23 (21.9%)	82 (78.1%)	0.158
Source of information is the government	2 (28.6%)	5 (71.4%)	1.000

### 3.6. Multivariate Analysis and the Correlates of Regular Influenza Vaccination

The results of the multivariate analysis can be found in Table 5. We conducted a multivariate analysis and found in model 1, that elderly people (OR = 2.25, CI = 1.08–4.71), with higher education (OR = 1.42, CI = 1.09–1.84), higher physical activity (OR significantly above 1), and chronic respiratory disease (OR = 3.24, CI = 1.58–6.62) are more regularly vaccinated, while those who visit the doctor only when needed (OR = 0.55, CI = 0.34–0.88) and those who consume more than 7 drinks/week (OR = 0.24, CI = 0.09–0.65) were less regularly vaccinated. Other factors, such as gender, residential area, alcohol consumption, financial situation, having health insurance had no influence on the probability of vaccine uptake.

**Table 5:** Multivariate analysis: correlates of regular influenza vaccination.

Model	Correlates of Regular Influenza Vaccination	OR	95% CI	*p*-Value
1	Age > 65 years	2.25	1.08–4.71	0.031
	Higher level of education	1.42	1.09–1.84	0.008
	Alcohol consumption (>7 drinks/week *vs.* never)	0.24	0.09–0.65	0.005
	Physical activity level			0.003
	Less than twice/week *vs.* never	1.85	1.13–3.04	0.015
	2–3 times/week *vs.* never	2.46	1.48–4.11	0.001
	More than 3 times/week *vs.* never	2.31	1.17–4.56	0.016
	Medical visits			0.004
	Once/year *vs.* routinely	1.15	0.63–2.09	0.650
	When needed *vs.* routinely	0.55	0.34–0.88	0.013
	Having a chronic respiratory disease	3.24	1.58–6.62	0.001
2	Medical visits			0.035
	Once/year *vs.* routinely	4.43	0.49–40.11	0.186
	When needed *vs.* routinely	0.30	0.06–1.61	0.162
	Thinking the vaccine is not needed	0.15	0.03–0.71	0.017

Model 1: In the stepwise descendent logistic regression to predict regular influenza vaccination, variables entered on step 1 were: gender, age, education, health insurance, financial situation, smoking, alcohol consumption, exercise, medical visits, heart disease, diabetes mellitus, kidney disease and respiratory disease. Nagelkerke R-Square = 0.143; Hosmer-Lemeshow: *p* = 0.417. Model 2: In addition to variables previously retained in model 1, knowledge and attitude variables were introduced. Nagelkerke R-square = 0.406; Hosmer-Lemeshow: *p* = 0.909.

When introducing knowledge and attitude variables to the previous model, the results showed “thinking that the vaccine was not needed” was the only correlate that demonstrated a significant inverse association with regular influenza vaccination (OR = 0.15; *p* = 0.017), while “visiting the doctor only when needed” showed an inverse but not significant association; all other variables were removed from the model because they were not significantly associated with regular influenza vaccination. 

## 4. Discussion 

### 4.1. Rates of Influenza Vaccination

The results from our study revealed an overall vaccination rate of 27.6%. This coverage might be an overestimation of the actual prevalence of vaccination in the Lebanese adult population since our subjects were interviewed in community pharmacies. Low vaccination rates are a worldwide public health problem. Continuous efforts to design and implement quality improvement interventions for increasing the rates of influenza vaccinations are being examined across nations. From patient reminder and recall systems to posters in physician offices tracking vaccination progress, no single intervention was able to solve this challenge [11,12,13]. 

Indeed, the National Internet Flu Survey sponsored by the CDC estimated a vaccination rate of the U.S adult population equivalent to 39.7% for the 2014–2015 influenza season [7]. European countries are also struggling to achieve the influenza vaccination specific targets as per the recommendations [8].

Low vaccination coverages become particularly concerning in high risk groups. In our study, elderly patients and/or patients with chronic diseases (heart disease, cancer, diabetes) or immunosuppressed statuses were also found to have suboptimal immunization rates ranging from 18.2% to 35%. For respiratory diseases, rates were improved to 50%, however still remained suboptimal. The European CDC recommended that influenza vaccination rates for winter 2014–2015 reach a target of 75% in older adults as well as in individuals with chronic conditions [8]. 

### 4.2. Factors Associated with Seasonal Influenza Vaccine Uptake

Future vaccination efforts have to particularly target subjects with low vaccine uptake. It is therefore important to recognize individuals who abstain from or oppose to receive the vaccine. Both our bivariate and multivariate analysis revealed that individuals with higher levels of education with active, non-sedentary lifestyles, non-smokers or heavy smokers, in addition to older adults (above 65 years) were positive predictors of vaccine uptake. Physically active, non-smokers, more educated individuals tend to perceive health as a priority and are able to make healthier life choices by adhering to recommendations [14].

These findings echo the results of many studies which found a strong association between low socio-economic status (SES) individuals and low vaccination rates. Indeed, Winston *et al.* documented a negative association between low SES subjects and seasonal influenza vaccination [15]. Additionally, Baudier and Leon observed much lower rates of up-to-date immunization profiles among the unemployed and those with lower educational levels [16]. The explanation to such findings can be a result of misinformation and ignorance in the low SES group [17,18]. Indeed, when examining the knowledge and attitudes of this group of participants, a clear lack of awareness on influenza disease symptoms, risks, vaccine availability, efficacy and safety was observed. 

An important additional contributing factor is the accessibility to vaccination. In fact, the majority of patients with a difficult financial situation stated that they thought the vaccine was expensive: 53.6%, *versus* 10.8% in case of manageable financial situation, and 3.4% for those with comfortable financial situation (*p* < 0.001; results not shown). This factor was however overridden in the multivariable analysis by the perceived need of the vaccine. 

Among patients with chronic conditions, only those with respiratory disorders were being immunized against influenza virus with statistical significance (*p* < 0.01), when compared to other patients suffering from other chronic diseases. This finding highlights the need to increase immunization efforts in the chronic disease population since influenza vaccine was proven to reduce hospitalization, morbidity and mortality in the elderly as well as the chronically ill patients [1,2,3]. For instance, vaccination in elderly was shown to decrease the risk of death from pneumococcal diseases and influenza-related complications by 50% and 80% respectively [4]. Furthermore, routine vaccination for diabetic children and adults was proven to reduce diabetes-related hospital admission by around 79% [19].

### 4.3. Knowledge and Attitude towards Vaccination

Our results revealed that “thinking that the vaccine was not needed” was the only correlate significantly associated with abstinence from regular vaccination. Clear misinformation exists among the Lebanese community towards the influenza vaccine. The population represented in our study falsely believed that regular influenza immunization is not essential. This evidence underscores a compelling need to raise public awareness regarding the importance of regular influenza vaccination.

### 4.4. Source of Vaccine Information

For all our studied participants, the main sources of information regarding vaccination were the physician and the pharmacists. Moreover, individuals who regularly followed-up with their physicians benefited from the vaccination opportunities offered during routine health care visits. The role of healthcare providers in promoting and reinforcing adherence to vaccination is clearly highlighted by this observation. Other studies also suggested that healthcare providers’ recommendations for regular vaccination were positive predictors to compliance [20,21]. Therefore, physicians, pharmacists, among other health care providers are invited to demonstrate knowledge of current immunization practices and recommendations. Remarkably, the role of the government, as a source of information, was revealed absent in the Lebanese community. The Lebanese ministries of health and public health should be prompted to educate the public on the importance of vaccination as well as to address the barriers to vaccine accessibility. For instance, adopting the influenza vaccine under the national vaccination program, organizing awareness campaigns and allocating national funds to cover it are essential steps.

### 4.5. Limitations

Our study does have potential limitations. Like all pharmacy-based patient surveys, a selection bias might have occurred. Extrapolation of data may not be accurate in institutionalized or homebound subjects or those individuals less likely to visit the pharmacy. Additionally, under-or inaccurate reporting might have occurred since participants were asked to self-report their vaccination statuses and awareness variables were measured using binary outcomes. The timing of the study might have influenced the results since participants might be more aware of influenza vaccination around fall and winter. Despite the mentioned limitations, the major strength of our study resides in the fact that it was the first one to include a large number of individuals surveyed from the general population across the country and report on their seasonal vaccination attitudes and practices.

## 5. Conclusions

The present study is the first to report the rates of vaccination among a selected Lebanese ambulatory adult population. Results showed that seasonal influenza immunization rates were low particularly among the low SES individuals. Patients with chronic diseases, other than respiratory, are sub optimally vaccinated. The major barrier to abstinence from vaccination is that the vaccine was viewed as not needed. The current results therefore emphasize the compelling need for spreading public awareness in Lebanon regarding the efficacy and benefits of seasonal influenza vaccination. Routine consultation with HCP is essential to enhance immunization opportunities. Furthermore, a collaborative effort of governmental bodies and health-care providers is recommended to educate, regulate and improve immunization practices through public and professional awareness campaigns.
